# Genome instability and crosstalk with the immune response

**DOI:** 10.1186/s13073-025-01509-6

**Published:** 2025-11-04

**Authors:** Roman M. Chabanon, François-Xavier Danlos, Kaissa Ouali, Sophie Postel-Vinay

**Affiliations:** 1 The ERC (Epi)genetic Vulnerabilities in Solid Tumors and Sarcoma Laboratory, Inserm Unit U981, Université Paris Saclay, Gustave Roussy, Villejuif, France; 2 The CRUK Gene Function Laboratory and Breast Cancer Now Toby Robins Breast Cancer Research Centre, The Institute of Cancer Research, London, UK; 3 Drug Development Department (DITEP), Gustave Roussy, Villejuif, France; 4 Department of Medical Oncology, Dana Farber Cancer Institute, Harvard Medical School, Boston, USA; 5 University College of London, Cancer Institute, London, UK; 6 Department of medical oncology, Gustave Roussy, Villejuif, France

**Keywords:** DNA damage response, Genomic instability, Replication stress, Aneuploidy, Cytoplasmic nucleic acid, Tumour immunogenicity, Anti-tumour immune response

## Abstract

Genome instability, tumour-promoting inflammation, and immune escape are three distinct hallmarks of cancer. However, accumulating scientific and clinical evidence over the past decade have uncovered a multifaceted interplay of complex dynamic network of interactions between genome instability, the DNA damage response (DDR), and tumour immunogenicity. Fuelled by the clinical successes of immune checkpoint blockers (ICB), growing interest for immuno-oncology and recent cancer biology discoveries have allowed a better understanding of the underlying biology and clinical opportunities brought by this interplay—which is yet, still only in its infancy. The cooperative nature of tumour cell-intrinsic and -extrinsic mechanisms involved suggests that harnessing genomic instability in cancer does not only hamper cancer cells fitness but also stimulate the anti-tumour immune response, thereby paving the way to the development of DDR-based immunomodulatory therapeutic strategies applicable to a variety of molecular and histological cancer types. Here, we review the various aspects of this crosstalk between genome instability and tumour immunogenicity, including feedforward and feedback mechanisms affecting either side of this interplay, as well as the specific consequences of chromosomal instability. We further discuss emerging DDR-based predictive biomarkers of response to ICB therapies, and finally examine the latest clinical developments of therapeutic combinations that exploit the DDR–immunity interplay in immuno-oncology.

## Background

Genome instability, which mostly results from defects in the DNA damage response (DDR), DNA replication, or cell division processes, was one of the first described hallmarks of cancer [1]. It has for long been known to underlie carcinogenesis, oncogenic transformation, and subsequent tumour progression. Over the past decade, mounting scientific and clinical evidence has shown that, beyond threatening genome integrity, genomic instability also influences several aspects of the anti-tumour immune response, both through cell-autonomous and non-cell-autonomous mechanisms which operate at the tumour–immunity interface [2–4].

Genome instability notably influences three major components of the DDR–anti-tumour immune response crosstalk: antigenicity, adjuvanticity, and reactogenicity [2]. The first of these components, antigenicity, relates to the ability of tumour cells to present immunogenic neoepitopes to cytotoxic or helper T-cells. By increasing mutability, genome instability favours the random formation of potentially immunogenic neoepitopes, which subsequently represent candidate neoantigens when presented at the cell membrane by major histocompatibility complexes (MHC), either by the tumour cell itself or via cross-presentation by an immune cell. The relevance in cancer of such mechanism has been best illustrated by the remarkable efficacy of anti-programmed death (ligand) 1 (anti-PD-(L)1) immune checkpoint blockers (ICB) in mismatch repair-deficient (MMRd), microsatellite-instable (MSI-H) tumours, first in the metastatic setting [5–8], and most recently in the adjuvant or neo-adjuvant settings [9, 10]. Most importantly, in 2020, anti-PD-(L)1 agents were the first ever drugs approved in a histotype-agnostic fashion, based on tumour mutational burden-high (TMB-H) as predictive biomarker [11]. This concretely showed case that primarily DDR-related oncogenic processes (here increased mutability and antigenicity resulting from MMR defects) can be therapeutically targeted solely using immunotherapy, with profound and prolonged efficacy in patients whatever the tumour type [5–8]. Perhaps more remarkably, DDR-based biomarkers (MMRd, MSI-H, or TMB-H), remain the best predictive biomarkers of efficacy of anti-PD-(L)1 therapy for patient selection, overcoming any immune-based biomarker including PD-L1 expression or the presence of tertiary lymphoid structures (TLS) [7]. This interplay between mutability, MMR deficiency, and tumour immunogenicity is now well-described and has been extensively reviewed elsewhere [2, 12].

Interestingly, subsequent research discovered that antigenicity was not the only driver of sensitivity to anti-PD-(L)1 agents of MMRd tumours and that adjuvanticity also played a role [13, 14]. Adjuvanticity refers to the ability of tumour cells to attract and/or activate immune cells through the release in the tumour microenvironment (TME) of cytokines, chemokines, or other adjuvant molecules. “Viral mimicry”, cytosolic nucleic acid sensing, and subsequent type I interferon innate immune response are essential components of adjuvanticity, which has traditionally been described as a “nucleus-to-cytoplasm” signalling process in cancer cells, for example following micronuclei formation and subsequent shedding of DNA or chromatin fragments in the cytoplasm [2, 15]. Intriguingly, very recent data show that some of the activated cytosolic immune pathways also reciprocally influence the replication and DDR machineries, suggesting the existence of a dynamic feedback loop that remains largely unexplored.

The third major component of the DDR–anti-tumour immune response crosstalk is known as reactogenicity, and describes the ability of tumour cells to modulate the activation of immune cells at the immunological synapse through co-stimulatory or co-inhibitory signals [2]. If this third component is far less described than antigenicity and adjuvanticity, it also represents a promising targetable platform which deserves further therapeutic exploration.

Finally, multiple processes that contribute to genomic instability (e.g. aneuploidy, DDR defects, replication stress, mitotic defects, or chromotripsis) can have opposite effects on tumour immunogenicity, thereby further complexifying this dynamic interplay [16–18].

Here, we review the most recent advances in our understanding of molecular mechanisms underlying the DDR–immunity interplay, notably those related to adjuvanticity where most novel, promising progresses are being achieved. We further present strategies and biomarkers that have been developed to translate such knowledge into the clinic, and discuss results of the latest corresponding clinical trials. We finally provide perspectives on future areas that could be further explored to best exploit this crosstalk as a therapeutic strategy.

## Genome instability: from DNA damage to immune signalling and beyond

### Cytosolic nucleic acid sensing mediates innate immunity in response to genome instability

It is now widely accepted that cytosolic nucleic acid sensing is a central component of innate immune responses, and a pivotal platform to stimulate anti-tumour immunity. The links between DDR, accumulation of cytosolic DNA, and activation of DNA-sensing-dependent innate immune signalling in cancer have been extensively reviewed elsewhere [2–4]. Here, we will shed light on the molecular mechanisms underlying those phenotypes, focusing on the consequences of replication stress and genome instability.

#### Replication stress as a source of cytosolic nucleic acids

Replication stress—which refers to a series of endogenous or exogenous events that perturb the replication machinery by altering the distribution of replication origins or impairing fork progression, is a known cause of genome instability and a hallmark of cancer cells. Replication stress is also a pivotal mechanism that links genome instability and cell immunogenicity. The proof-of-concept for this interconnection is best illustrated by the observation that inactivating mutations in genes maintaining DNA replication homeostasis—such as those encoding RNase H2, SAMHD1, or ADAR1—are pathognomonic of the Aicardi-Goutières syndrome (AGS) [19–23], a rare and severe neuroinflammatory disorder associated with systemic autoinflammation and elevated type I interferon expression. If the causes of AGS remain partially unclear, accumulating data suggest that the loss of these enzymes leads to an increased genomic R-loop burden, which favours replication stress, DNA damage, and activation of cell-autonomous innate immune responses [20, 24]. In line with this notion, AGS caused by *RNASEH2* mutations has been mainly attributed to genome instability and a p53-dependent DDR due to the accumulation of ribonucleotides in genomic DNA [25–27]. Importantly, other known genetic determinants of type I interferonopathies include deficiencies in other cytoplasmic DNA and RNA metabolism enzymes notably TREX1, STING, MDA5, and RIG-1 [28], further connecting the accumulation of cytosolic nucleic acids with the activation of AGS-associated immune responses.

#### Cytosolic DNA sensing

**Fig. 1:**
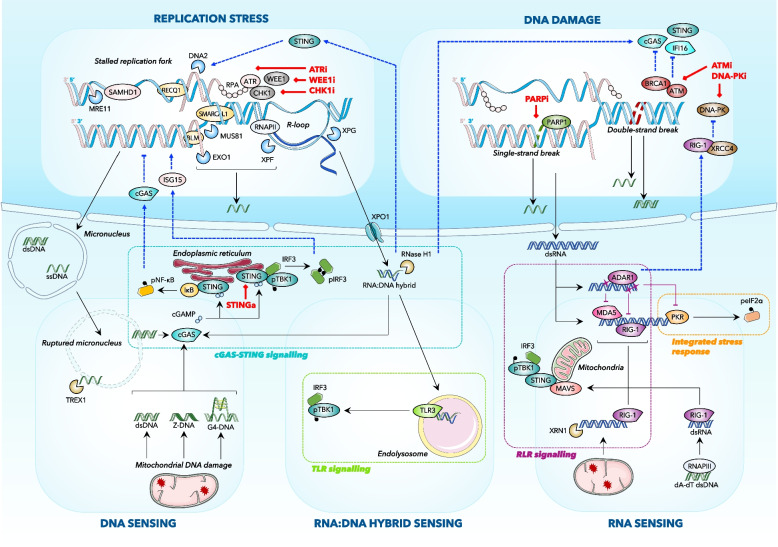
Dynamics of the mechanisms underlying the DNA damage–replication stress–cytosolic immunity interplay in cancer. Black arrows indicate nucleus-to-cytoplasm feedforward signalling. Dashed blue arrows indicate cytoplasm-to-nucleus feedback signalling. The molecular players involved in replication stress, DNA damage, and cytosolic nucleic acid sensing are highlighted in blue boxes and categorized by pathway: surrounded by dashed rectangles in the cytosol, cGAS-STING signalling (turquoise blue), TLR signalling (apple green), RLR signalling (fuchsia) and integrated stress response (orange); in the nucleus, HRR (red), NHEJ (brown), base-excision repair (olive green), replication stress response (pale pink), DNA endo- and exonucleases (sky blue), DNA helicases (yellow), cell cycle (grey). Pharmacologically actionable targets are highlighted with the corresponding clinical compounds in bold red. Gene or protein names are as follows, arranged by alphabetical order: 53BP1, Tumour Protein P53 Binding Protein 1; ADAR1, Adenosine Deaminase Acting on RNA 1; ATM, Ataxia Telangiectasia Mutated; ATR, Ataxia Telangiectasia And Rad3-Related Protein; BLM, Bloom Syndrome RecQ Like Helicase; BRCA1, Breast Cancer Type 1 Susceptibility Protein; CGAS, Cyclic GMP-AMP Synthase; DNA-PK, DNA-Dependent Protein Kinase Catalytic Subunit; DNA2, DNA Replication Helicase/Nuclease 2; eIF2α, Eukaryotic Translation Initiation Factor 2A; EXO1, Exonuclease 1; IFI16, Interferon Gamma Inducible Protein 16; IkB, Inhibitor of Nuclear Factor Kappa B Kinase Subunit Beta; IRF3, Interferon Regulatory Factor 3; ISG15, Interferon-Stimulated Protein 15 kDa; LGP2, DExH-Box Helicase 58; MAVS, Mitochondrial Antiviral Signalling Protein; MDA5, Melanoma Differentiation-Associated Protein 5; MLKL, Mixed Lineage Kinase Domain Like Pseudokinase; MRE11, Meiotic Recombination 11 Homolog, Double Strand Break Repair Nuclease; MUS81, MUS81 Structure-Specific Endonuclease Homolog; NF-kB, Nuclear Factor Kappa B; PARP1, Poly(ADP-Ribose) Polymerase 1; PKR, Protein Kinase R; Polα/δ/ε, DNA Polymerase Alpha / Delta / Epsilon; RECQ1, RecQ Like Helicase; RIG-1, Retinoic Acid-Inducible Gene 1 Protein; RIPK1/3, Receptor Interacting Serine/Threonine Kinase 1/3; RNAPII/III, RNA Polymerase II/III; RPA, Replication Protein A; SAMHD1, SAM And HD Domain Containing Deoxynucleoside Triphosphate Triphosphohydrolase 1; SMARCAL1, SWI/SNF Related, Matrix Associated, Actin Dependent Regulator of Chromatin, Subfamily A Like 1; STING, Stimulator of Interferon Genes Protein; TBK1, TANK Binding Kinase 1; TLR3, Toll Like Receptor 3; TRAF3/6, TNF Receptor Associated Factor 3/6; TRIF, Toll Like Receptor Adaptor Molecule 1; XPF/G, Xeroderma Pigmentosum Complementation Group F/G; XPO1, Exportin 1; XRCC4, X-Ray Repair Cross Complementing 4; ZBP1, Z-DNA Binding Protein 1. Abbreviations: cGAMP, 2'3'-cyclic GMP-AMP; dA-dT dsDNA, Poly(Deoxyadenylic-Deoxythymidylic) Double-Stranded DNA; dsDNA, Double-Stranded DNA; dsRNA, Double-Stranded RNA; G4-DNA, G-Quadruplex DNA; PAR, Poly(ADP-Ribose); RLR, RIG-1 Like Receptor; ssDNA, Single-Stranded DNA; TLR, Toll Like Receptor; Z-DNA, Z-form DNA

Multiple endogenous or exogenous sources of replication stress have proven to stimulate cytosolic DNA accumulation and cGAS-STING-mediated innate immunity, including nucleotide pool imbalance [29, 30], oncogene-induced abnormal origin firing [31], R-loops [32–35], cellular ageing [36], stemness [37], and unrepaired DNA damage caused by exposure to DNA-damaging agents [38–45], DDR-targeted therapies—including inhibitors of Poly(ADP)-ribose Polymarase (PARP) [15, 46–50], Ataxia-Telangectasia-related ATR [51–54], ATM [55–57], WEE1 [58], POLQ [59], or epigenetic enzymes [60]. In cancer, the function of enzymes mediating the resolution (via repair or degradation) of stalled and/or damaged replication forks has been linked to processes that control cell-autonomous innate immune responses (Fig. 1) [2]. In particular, the activity of structure-specific endonucleases (such as MUS81, which mediates cleavage of DNA structures at stalled replication forks; or XPF and XPG, which mediate processing of unscheduled or persistent R-loops) and exonucleases (e.g. EXO1, which facilitates the resolution of Holliday junctions; or MRE11, which mediates degradation of nascent DNA at stalled replication forks) has been shown to promote the accumulation of cytosolic DNA fragments that become immunogenic upon sensing by the cGAS-STING pathway [20, 21, 45, 61, 62]. Of note, impaired protection of ssDNA during replication was also associated with cGAS-STING pathway activation in the contexts of RPA, RAD51, or FANCD2 deficiency [63–65], highlighting the key role of nucleases in generating immunogenic nucleic acids upon replication stress. Similar processes also operate in the context of mitochondrial genome (mtDNA) instability, suggesting a common functional origin for the diverse forms of replication stress-driven cell-autonomous innate immunity [66]. Interestingly, the annealing helicase SMARCAL1—which catalyses replication fork reversal and Holliday junction migration to maintain genome stability during replication [67–69], was shown to suppress cGAS-STING signalling in cancer cells by limiting endogenous DNA damage [70], while the structure-specific helicase BLM—which also mediates Holliday junction resolution [71], has conversely been shown to promote [45] or prevent [72] the accumulation of immunogenic DNA fragments in the cytosol, suggesting a double-edged sword feature of DNA helicases.

Importantly, the cGAS-STING pathway is known to mediate several biological processes beyond type I interferon induction, including noncanonical autophagy via LC3B lipidation [73], and lysosomal cell death via NLRP3 inflammasome activation [74]. These downstream processes have recently been linked to the proton channel function of STING [75], which mediates proton leakage from the Golgi apparatus into the cytoplasm to stimulate both LC3B lipidation and NLRP3 activation. Whether and how these mechanisms operate in the face of genome instability remains to be explored; still, considering the role of autophagy and inflammasome activation in inducing immunogenic forms of cell death [76], one might expect an overall immunostimulating interaction of the latter mechanisms with cytosolic nucleic acid sensing PRR activation.

#### Cytosolic RNA sensing

If the accumulation of cytosolic DNA resulting from genome instability is a well-appreciated, potent immunogenic adjuvant in cancer cells, other cytosolic nucleic acids have also been shown to contribute to tumour adjuvanticity via the activation of specific pattern recognition receptors (PRR; Fig. 1).

Immunogenic RNA species can arise in the cytoplasm as a result of DNA damage or replication stress, and stimulate interferon signalling notably via RIG-1-like receptors (RLRs) RNA sensing pathways [77]. RLRs were shown to be essential for the induction of cell death following DNA double-strand breaks (DSBs) [78], suggesting RIG-1-mediated innate immune signalling contributes to immunogenic cell death in response to DNA damage. In particular, RIG-1-mediated innate immune responses have been observed in the context of DNA damage or replication stress elicited by anti-cancer therapies including chemotherapy [79, 80], radiotherapy [79, 81, 82], DDR-targeted therapies [51, 83, 84], and epigenetic modulators [85–89]. The mechanisms underlying such responses remain poorly understood but the current literature allows to consider that (i) cytosolic poly(dA-dT) DNA can be converted into 5'-ppp dsRNA via RNAPIII-dependent transcription to activate RIG-1 [51, 90]; (ii) cytosolic RNA can accumulate following transcriptional upregulation (or de-repression) of non-coding RNA species, notably small non-coding RNAs [81] or transposable elements, including interspersed nuclear elements (SINEs and LINEs) [79, 80] and endogenous retroviruses (ERVs) [82, 87, 88]. Interestingly, cytosolic mitochondrial RNA (mtRNA) can also be released as a result of mtDNA damage [91], a mechanism that does not necessarily coincide with cell death.

Corroborating these findings, a very recent study showed that pharmacological induction of chromosome mis-segregation caused an accumulation of dsRNA-forming transcripts derived from intergenic and intronic regions of DNA captured in micronuclei, including sequences from interspersed nuclear elements and ERVs [92]. Activation of cytosolic dsRNA sensing in this context was shown to cooperate with cGAS/STING-mediated dsDNA sensing, stimulating immune cell migration and cancer cell antigen presentation.

#### Cytosolic RNA:DNA hybrids sensing

RNA:DNA hybrids—referred to as R-loops when stably formed in genomic dsDNA where they displace one DNA strand to generate a three-stranded structure, are physiological intermediates in DNA replication, repair, and transcription [93]. Yet, R-loops also represent a threat to genome stability, especially during DNA replication where transcription-replication conflicts can occur as a result of altered R-loop levels [93]. In cancer, R-loops have been associated with replication stress [94–97], and a number of studies have showed that cytosolic ssDNA fragments can arise from this situation, causing the induction of interferon and inflammatory responses in a cGAS-STING-dependent manner [32–35].

Interestingly, few recent lines of evidence also suggest that a subset of R–loop-derived RNA:DNA hybrids could accumulate in the cytosol as a result of perturbed genomic R-loop burden [98], leading to PRR-dependent innate immune responses [98, 99]. Cytosolic RNA:DNA hybrids were shown to derive from a restricted subset of relatively long-lived genomic R-loops that are partially RNase H resistant, and form at regions of convergent transcription (i.e. sites in which both sense and antisense R-loop form in a head-on orientation) harbouring nucleotide skew [98]. This population of cytosolic RNA:DNA hybrids was shown to be sensitive to XPF/XPG silencing and pharmacological inhibition of the nuclear transport receptor XPO1, suggesting that these hybrids likely originate from the nucleolytic processing of genomic R-loops and are actively exported to the cytoplasm [98]. Intriguingly, cGAS and the ubiquitous PRR TLR3 (whose canonical activation has been ascribed to DNA and RNA, respectively)—and to a lesser extent RIG-1 or MDA5, were shown to mediate RNA:DNA hybrids sensing, which raises the question of the structural specificity, compartmentalization, and potential cell type specificity of PRR-mediated hybrids detection. These findings nonetheless establish cytosolic RNA:DNA hybrids as a novel immunogenic population of nucleic acids that aberrantly accumulate in the context of genome instability and participate to tumour immunogenicity.

#### Sensing of non-canonical forms of cytosolic nucleic acids

Few but worthy of note studies have more recently uncovered the potential of non-canonical DNA and RNA structures to elicit cytosolic immunity in the context of genome instability or DNA damage, notably: (i) cytosolic G-quadruplex DNA (G4-DNA), which accumulates in response to oxidative stress [100] or mtDNA damage elicited by G4-ligands [101], leads to cGAS-STING pathway activation; (ii) cytosolic Z-form DNA (Z-DNA), which accumulates as a result of chemotherapy-induced mitochondrial genome instability [102], leads to an inflammatory response cooperatively mediated by ZBP1 and cGAS [102, 103]; (iii) cytosolic Z-form RNA (Z-RNA), including Z-RNA Alu-Alu duplexes, strongly induces ZBP1-dependent cell death upon ADAR1 loss [104–106]; and (iv) cytosolic telomeric-repeat-containing RNA (TERRA) transcripts, which are synthesized from dysfunctional telomeres in the context of replicative crisis, amplify via ZBP1 activation a cGAS-STING-mediated interferon response initiated by the sensing of broken telomere ends, driving cell death in concert with autophagy [107].

### Cytosolic nucleic acid sensing in anti-tumour immunity: a complex and interconnected network

Relationships between nucleic acid sensing and the resulting anti-tumour innate immune response are complex due to crosstalk and redundancy between nucleic acid sensing pathways, which can occur concomitantly or sequentially. First, several PRR are redundant for the detection of certain templates (e.g. dsRNA sensing mediated by over 25 different proteins) and share common signal transducer molecules (e.g. TBK1 phosphorylation resulting from cGAS, TLR3, RIG-1, or ZBP1 activation). Second, these are highly subjected to self- and cross-regulation (e.g. positive cooperation of TLR and inflammasome signalling pathways) [108]. Third, DNA and RNA sensing PRR can cooperate to stimulate innate immunity in response to genomic insults [62, 107]: the observation that interferon signalling following SAMHD1 mutation is driven by the MDA5-MAVS pathway but requires functional priming through the cGAS-STING pathway is an example of this interdependence [62]. Fourth, context-specific mechanisms have emerged, such as for instance, the predominance of ssDNA sensing in MMRd tumour cells [13, 14]. Fifth, non-canonical species (e.g. cytosolic RNA:DNA hybrids [98, 109, 110]) and converted forms of nucleic acids (e.g. transcribed DNA or reverse-transcribed RNA, see examples below) were also found to be immunogenic when present in the cytosol, potentially contributing to the activation (simultaneously or sequentially) of several distinct PRR pathways. Altogether, this renders the exact determination of the contribution of each PRR pathway to a given phenotype extremely challenging. Still, it appears that TBK1 phosphorylation, followed by IRF3 phosphorylation and its subsequent nuclear translocation, represents a key signalling node that drives innate immune responses following the activation of many nucleic acid sensing pathways.

Beyond their formation and release into the cytosol, parameters that also influence the accumulation of cytosolic nucleic acids are their metabolism and half-life, which notably depends on their rate of degradation and/or conversion. The metabolic turnover of cytosolic nucleic acids is primarily controlled by the well-described cytosolic activity of various nucleases, including but not limited to the DNA-degrading exonuclease TREX1, RNA-degrading enzymes XRN1 [111] and RNase L [112], or the RNA:DNA hybrid-degrading enzymes RNase H1 and RNase H2, which may be active in the cytosol [113]. Defects in these enzymes have been associated with inflammatory phenotypes [114–117], highlighting the fact that their undegraded substrates constitutively activate PRR-mediated innate immune responses. Importantly, cytosolic nucleic acids can also be converted into different PRR-activating species in the cytosol, as exemplified by the RNAPIII-mediated conversion of cytosolic poly(dA-dT) DNA into 5′-ppp RNA capable, which causes RIG-1 activation [90], and the reverse transcription of ERV-derived cytosolic dsRNA into dsDNA, which provides a substrate for cGAS-STING activation [118] or may favour the accumulation of immunogenic RNA:DNA hybrids [119]. All these mechanisms illustrate how the complex and dynamic nature of cytosolic nucleic acid metabolic regulation impacts on innate immune responses.

Finally, although most studies support the idea that activation of cytosolic nucleic acid sensing PRR enhances anti-tumour immunity, opposing effects have also been described in some contexts. In particular, chronic engagement of the cGAS-STING pathway was shown to cause pro-tumourigenic effects via senescence evasion [120], promoting metastasis and treatment resistance in some preclinical models [17, 118]. Similarly, RIG-1 agonism mediated by unshielded, RNA-binding protein-free, RNA transcripts from stromal exosomes was shown to cause an inflammatory response enhancing tumour growth, metastasis, and therapy resistance in breast cancer models [121]. These studies underline the potential double-edge sword nature of cytosolic nucleic acid sensing responses in cancer.

### Cytosolic immune signals reciprocally affect genome stability

An emerging body of literature now suggests that cytosolic nucleic acid sensing and the resultant innate immune responses reciprocally affect genome stability and the DDR via multiple cytoplasm-to-nucleus signalling axes (Fig. 1).

#### cGAS, STING, and ISG15 are modulators of replication forks

Two important observations recently provided the first hints of a reciprocal interplay between the DDR and cytosolic immunity: (i) cytosolic DNA sensors can be found inside the nucleus (e.g. cGAS, IFI16 [122–127])—which suggests a potentially compartmentalized function of PRR; and (ii) multiple canonical DNA repair and replication proteins (e.g. MRE11, DNA-PK, DHX9, etc. [108]) can serve as PRR in the cytosol.

The regulation of replication fork velocity and nascent DNA synthesis by cGAS, STING, and ISG15 provides a salient illustration of the reciprocity of this dynamic genome instability–immune response crosstalk, which allows an adaptive and coordinated systemic cellular response to DNA damage. In the nucleus, chromatin-bound cGAS was found to act as a decelerator of replication forks, preventing genome instability and replication-dependent DNA damage [128]. Likewise, STING activation was found to promote calcium ions-dependent replication fork protection in response to replication stress—a physiological mechanism involving activation of CAMKK2/AMPK via increased intracellular levels of Ca^2+^, preventing uncontrolled fork processing by EXO1 [129]. Intriguingly, replication stress-dependent STING activation was also associated with increased nascent DNA degradation at stalled replication forks [130], suggesting a potential double-edged sword effect of cGAS-STING pathway activation on genome integrity. Similarly, ISG15—an interferon-stimulated protein upregulated in response to cGAS-STING pathway activation, was early identified as a partner of PCNA and an important mediator in the termination of error-prone translesion DNA synthesis [131]. ISG15 was initially shown to facilitate replication fork restart via a non-covalent functional interaction with RECQ1 [132], and to protect nascent DNA from degradation upon replication stress, via the ISGylation of fork-interacting proteins that promote its stabilization, including TOP1 [133], TOP2A, and FEN1 [134]. Conversely, ISG15-mediated acceleration of replication forks was recently shown to increase replication stress and chromosomal instability in cancer cells [132], highlighting the potentially dual effects of ISG15 on DNA replication.

The functional interaction between cGAS, MRE11, and chromatin further illustrates the complexity of this interplay. Indeed, if structural studies initially described that cGAS binding to the nucleosome acidic patch sterically hindered its activation by dsDNA –acting as a constitutive lock against cGAS/STING activation [123, 124], more recent data indicate that MRE11 binding to cytosolic chromatin fragments could displace cGAS from nucleosomal-mediated sequestration, thereby enabling its mobilization and activation by dsDNA [135].

Altogether, these examples illustrate the dynamic, multilevel regulations at stake and highlight a previously unrecognised complexity of the DDR–cytosolic immunity interplay, which may have pleiotropic effects on genome stability and the anti-cancer immune response.

#### cGAS, STING, RIG-1, and IFI16 are modulators of the DNA damage response

In addition to their impact on DNA replication, recent evidence supports that cytosolic nucleic acid sensors directly modulate the DDR, here again with potentially opposite effects. cGAS–STING pathway activation was found to promote DDR signalling in a STING- and TBK1-dependent manner [136], notably via the stimulation of ATM- and p21-mediated cell cycle arrest [137, 138] or deoxyribonucleotide metabolism [139]. At telomeres, cGAS-mediated suppression of mitotic DNA DSB repair also protected mitotic chromosomes from end-to-end fusions, thereby preserving genome stability by facilitating replicative senescence [140]. Conversely, cGAS was shown to translocate to the nucleus upon DNA damage [141] and inhibit homologous recombination repair (HRR) at sites of DNA DSBs [141, 142], hence enhancing genome instability. IFI16 was also shown to suppress DNA DSB repair by hampering ATM signalling and the subsequent recruitment of repair proteins to damage sites, including γ-H2AX, HRR (BRCA1, 53BP1, and NBS1) and non-homologous end joining (NHEJ) factors (DNA–PK) [143]. Similarly, the dsRNA sensor RIG-1 was shown to specifically suppress NHEJ at sites of DNA DSBs via interaction with XRCC4, competitively preventing the formation of the XRCC4/LIG4/XLF complex at DSBs [144]. These examples highlight an interesting paradigm: while the suppression of DSB repair pathways by cytosolic nucleic acid sensors is advantageous upon viral infection by protecting cells against integration of the viral DNA in the genome, these same effects will be detrimental and further hamper genome instability in the context of DNA damage or replication stress in cancer.

### Tumour cell aneuploidy affects cancer–immune cells interactions

Aneuploidy refers to an abnormal number of chromosomes in a cell. It often arises as a result of chromosomal instability (CIN), which itself can be the consequence of DDR defects [145, 146]. Altogether, these abnormalities not only contribute to tumour heterogeneity and adaptability, but also disrupt normal cell cycle control, apoptosis, and notably, shape the TME [147].

#### Opposing effects of chromosomal instability on the tumour immune microenvironment

Aneuploidy-derived structural abnormalities of chromosomes can favour chromothripsis and the subsequent formation of micronuclei [148], thereby triggering the cGAS-STING pathway (Fig. 1) [38, 39, 146, 149]. Indeed, cells with pre-existing CIN were shown to exhibit elevated levels of micronuclei, which activate cGAS when dsDNA becomes accessible upon micronuclei envelope disruption [17]. The resultant production of type I interferons and associated cytokines is traditionally known to enhance host immune surveillance [150, 151], acting as an alarm signal that rewires the tumour immune microenvironment to a more active state, via increased cytotoxic activity of CD8 + T-cells and NK cells against malignant cells, activation of dendritic cells, and enhanced presentation of tumour antigens to T-cells following MHC upregulation [152, 153].

Yet, paradoxically, aneuploid tumours tend to exhibit reduced immunogenicity or decreased immune cell infiltration [154, 155]. Notably, chronic cGAS/STING-mediated type I interferon production was shown to stimulate tumour progression and dissemination, via cancer cell-intrinsic and -extrinsic effects [17, 156, 157]. It was first shown that chromosome segregation defects in the context of CIN could cause a deleterious, metastasis-promoting tumour cell-autonomous response to cytosolic DNA via the activation of non-canonical NF-κB signalling and inflammatory pathways downstream of STING—to the detriment of a type I interferon response [17]. Similarly, CIN was shown to trigger an inflammatory IL-6/STAT3-mediated signalling axis via cGAS/STING, thereby exerting autocrine pro-tumoural effects that can be restrained by IL-6 receptor blockade [158]. More recently, a single-cell transcriptomic study showed that CIN-induced chronic activation of the cGAS/STING pathway could result in the rewiring of downstream signalling in cancer cells, leading to a pro-metastatic TME via type I interferon tachyphylaxis (i.e. the reduction in interferon responsiveness to repetitive cGAS/STING stimulation) and an increased cell-autonomous endoplasmic reticulum (ER) stress response [157]. Thus, as opposed to the well-known anti-tumour effects of type I interferons, sustained cGAS/STING pathway stimulation in aneuploid tumour cells leads to pro-inflammatory responses manifested by signalling molecules that act as tumour growth or invasion-promoting factors [158].

By contrast, aneuploid cells also often exhibit a senescence-associated secretory phenotype (SASP) characterized by the release of pro-inflammatory cytokines, chemokines, and growth factors such as macrophage inflammatory protein (MIP)−3α and −1α (CCL20, CCL3), monocyte chemoattractant protein (MCP)−1, −2 and −4 (CCL2, CCL8, and CCL13), and eotaxin-3 (CCL26) [145, 159]. These secreted factors influence both tumour and immune cells [160], creating a dynamic interplay that can either favour or inhibit tumour progression [160, 161]. Aneuploid cells can also express stress-induced ligands recognized by NK cells, which play a crucial role in the anti-tumour innate immune response by eliminating cells that exhibit signs of stress or transformation [162].

Beyond these aspects, the clinical trajectory of cancers with elevated CIN is often complicated by their ability to evade immune surveillance, a challenge further exacerbated by immuno-editing where an immune selective pressure is at play, inducing immune escape mechanisms [163–167]. Such cancers define a highly aggressive subset of tumours, with metastatic potential, often resistant to standard therapies, including chemotherapy and radiotherapy [168]. This resistance is, at least in part, favoured by the genetic plasticity induced by aneuploidy, which confers tumour cells with the ability to rapidly adapt to diverse and hostile environments. Therefore, if adaptive immune responses dominated by interferon-γ-expressing T-cells can initially contain tumour growth [169, 170], tumour cells eventually exploit genomic instability to lose cell growth regulators and escape immune surveillance, which challenges treatment strategies [171]. For example, deletions involving the 9p21 region, which lead to *CDKN2A* loss and concurrent loss of type I interferon genes, have been linked to immune evasion and tumour progression [172]. Along similar lines, a recent study of the mutational landscape of metastases in patients with cutaneous melanoma refractory to BRAF inhibitors and ICB therapy [173] revealed that late mutational signatures were dominated by an accumulation of mutations in DNA repair pathways, as well as alterations affecting multiple immune-evasive processes (e.g., loss-of-function alterations in *CDKN2A*, *B2M*, *JAK2*, *CD274*/*PD*-*L1*, and *PTEN*), and associated with an immune desert TME [173]. Other copy number alterations impacting tumour immunogenicity include for instance Y chromosome deletions, which promote evasion from cytotoxic CD8 + T-cell elimination, causing increased tumour aggressiveness [174].

Altogether, these findings highlight the double-edge sword consequences of aneuploidy on the anti-tumour immune response, illustrating how diverse genetic alterations collectively shape the TME and influence clinical outcomes. This poses challenges that are inherent to chromosomally-instable cancers, underscoring the urgent need for dedicated, mechanism-based therapeutic strategies capable of harnessing the immune system to combat this subset of malignancies.

#### Context-specific spontaneous immune responses to chromosomal instability

Interestingly, immune responses can vary across different cancer types and immune cells adapt to tumour-intrinsic features of genomic instability or CIN—including the type and burden of DNA damage, antigen presentation capacity, (sub)clonal architecture, or local cytokine milieu, whose combination determines divergent outcomes across different tumour contexts. Notably, spontaneous immune responses to CIN do not occur uniformly across all cancer types: differences in magnitude of the response and qualitative composition of immune infiltrates have been described. Tumours such as melanoma, non-small cell lung cancer (NSCLC), and head and neck squamous cell carcinoma (HNSCC), which are typically TMB-H due to exogenous exposure to UV light, tobacco, or alcohol-driven mutagenesis, often display robust CD8 + T-cell infiltration and proinflammatory immune microenvironments [175–177]. These “hot” tumours are enriched in neoantigens and often harbour spontaneous T-cell responses directed against mutation-derived peptides. High-grade serous ovarian carcinomas (HGSOC) and triple-negative breast cancers (TNBC), some of which being associated with HR-deficiency (HRD)-driven genomic instability and cGAS-STING activation, are less frequently inflamed but show innate immune activation associated with the presence of lymphoid aggregates ranging from small, diffuse immune cells clusters to large, mature tertiary lymphoid structures (TLS) often surrounded by dense infiltrates of plasma cells [178–180]. By contrast, despite the presence of immunogenic stimuli such as neoantigens or cytosolic DNA fragments, tumours characterized by persistent CIN, complex aneuploidies or polyploidy resulting from whole-genome duplication often exhibit low levels of tumour-infiltrating lymphocytes (TILs). Such tumours notably include some TNBC, castration-resistant prostate cancer (CRPC), pancreatic or hepatobiliary cancers, anaplastic thyroid cancer, glioblastoma, esophagogastric or lung cancers, and poorly differentiated or osteosarcomas. In this context, chromosome copy number heterogeneity likely favours natural selection processes which enhance tumour fitness and facilitate mechanisms of immune exclusion or suppression [18] through tumour evolution, as previously highlighted.

Of note, organ-specific immune microenvironments apply to both primary tumours and metastases: notably, the immunogenicity of cancer metastases varies according to their location in the body due to tissue-specific microenvironmental characteristics, such as tissue-resident immune cells, local or regional cytokine signalling, and site-specific secondary tumour evolution [181]. For example, lung metastases are generally highly T-cell inflamed and more immunogenic than their bone, brain, or liver counterparts, regardless of the primary tumour of origin [182], whereas liver metastases are associated with systemic CD8 + T-cell depletion and worse outcome on anti-PD-1 immunotherapy [183, 184]. These examples highlight that beyond the primary tumour-intrinsic features of genomic instability or CIN, tissue-specific processes also determine metastatic dissemination and systemic immune responses to cancer progression.

Furthermore, regardless of the histotype, there is also substantial inter-patient variability in immune responses to genomic instability, resulting notably from differences in timing, extent, and nature of the underlying genomic alterations [185, 186]. Intra-tumour heterogeneity—which is favoured by ongoing CIN, genomic instability, and resulting clonal evolution—also leads to coexisting tumour subclones with distinct immunogenic profiles and immune escape mechanisms in a single patient [187, 188]. An illustration of this is subclonal loss-of-heterozygozity (LOH) of the HLA locus, an immune escape mechanism prevalent in many cancers including NSCLC, which frequently occurs following whole-genome duplication and is subject to strong microenvironmental selection pressures later in tumour evolution [186, 189]. These dynamics may explain the mixed and evolving immune landscapes observed in many tumours, which are shaped by their unique evolutionary genetic trajectory consisting of potentially co-occurring (or sequentially occurring) mechanisms favouring immune activation (e.g., via neoantigen production or cGAS-STING signalling activation) or immune suppression (e.g., via chronic inflammation, immunoediting, or activation of tolerogenic cytokine pathways such as IL-6/STAT3). Understanding tumour- and patient-specific patterns of immune adaptation, as well as their dynamic evolution, is therefore essential for customizing immunotherapeutic strategies to the genetic context [190].

## Emerging biomarkers exploiting the DNA damage response–immunity interface in immuno-oncology

### DNA repair defects as biomarkers of response to immune checkpoint blockade

Genome instability is a hallmark of DDR-defective tumours, but all DDR defects do not equally contribute to tumour immunogenicity. Notably, if deficiencies in MMR (including defects in MLH1, PMS2, MSH2, and MSH6 [5, 6, 191, 192]) and DNA proofreading polymerases (POLE [193–196] and POLD1 [193]) are known to drive MSI, hypermutability, elevated TMB, and a resultant increased tumour antigenicity [197–199], deficiencies in other DDR pathways appear to be less effective in doing so [200]. Further, and as opposed to MMR deficiency, the latter may be tumour type-dependent [200, 201]. For example, defects in HRR [200, 202–206], base-excision repair [207], or nucleotide-excision repair (NER) [208, 209] have been associated with increased TMB, tumour antigenicity, and TILs, to an extent that varies according to the altered pathway [201]. The correlation between increased TMB and better outcome upon ICB treatment has been retrospectively validated in patients with melanoma [210], NSCLC [193, 211, 212], urothelial bladder carcinoma (UBC) [213], and HNSCC [214]. Accordingly, TMB of ten or more mutations per megabase, MMR deficiency, and MSI-H are currently the only tumour-agnostic FDA-approved biomarkers of response to anti-PD-(L)1 therapy [5–7, 191]. Still, recent data supports that thresholds used to define TMB-H may be refined and customized according to the tumour type [215]. HRR defects have also been identified as predictive biomarkers of response to anti-PD-1 therapy but in a restricted set of tumour types [216–219], and much less potently than TMB-H.

### Beyond tumour mutational burden: additional genome instability-related biomarkers of response to immune checkpoint blockers

Response rates to anti-PD-(L)1 range from 30 to 50% in MSI-high tumours [6, 192], suggesting the presence of additional determinants of response. If variability in TMB may in part underlie these differences [197], impaired cytosolic immunity [13, 14], intra-tumour heterogeneity [220, 221], neoantigen clonality [222], co-occurring mutations [164], or unfavourable TME composition [223–225] can all drive resistance to ICB.

Cytosolic immunity has notably emerged as an important determinant of response to ICB [2]. In particular, the cGAS-STING pathway has proven to be essential for the anti-tumour effects of anti-PD-(L)1 in preclinical models [13, 14, 226], and higher expression of cGAS and STING correlated with improved response rates and survival of MLH1-deficient cancer patients treated with anti-PD-1 [13, 14]. Activation of the cGAS-STING pathway has further been described in tumours undergoing DNA damage caused by endogenous DDR defects [15, 41, 48, 64, 227–233], a phenotype that was linked to increased TILs, notably in the context of HRR and NER defects [15, 31, 41], suggesting that cGAS-STING-driven innate immune signalling in DDR-defective tumours could improve response to ICB by favouring an immunologically “hot” TME. Along similar lines, RIG-1 signalling was shown to be critical for systemic tumour control following anti-CTLA-4 therapy in preclinical models, and higher expression of RIG-1 correlated with prolonged survival and durable responses to anti-CTLA-4 in melanoma patients [234]. More recently, ADAR1 has been identified as a potential determinant of resistance to ICB due to the repression of MDA5- and PKR-mediated dsRNA sensing [235] or ZBP1-mediated Z-RNA sensing [236], which resulted in impairment of tumour inflammation, immune infiltration, and interferon-mediated tumour growth arrest, or RIPK3-mediated necroptosis of tumour cells, respectively.

Collectively, these examples underline the importance and clinical relevance of cytosolic nucleic acid sensors as potential predictive biomarkers of response to ICB, and further support their incorporation alongside immune-related biomarkers as a strategy to optimize patient selection. Very interestingly, a replication stress response (RSR) defects gene signature has recently been developed along this line. It showed a positive correlation with ICB response in 12 cohorts of patients with non-hypermutated cancers across seven tumour types [225]. In this study, RSR defects resulted in the accumulation of immunostimulatory cytosolic DNA and improved ICB response in preclinical models of breast and renal cell carcinomas. Besides providing a practical approach to predict response to ICB in TMB-low cancers, these findings suggest that pharmacological manipulation of the DDR and RSR could expand the benefits of ICB to a larger patient population (e.g. [145], reviewed in [2]).

### Genome instability-related biomarkers of tumour resistance to immune checkpoint blockade

Beyond tumour-intrinsic biomarkers associated with high TMB and a resultant increased immunogenic neoantigens repertoire, most identified biomarkers associated with anti-PD-(L)1 efficacy support the presence of a pre-existing adaptive immune response [237–241], suggesting that upregulation of immune-inhibitory pathways represent a central mechanism by which tumours escape immune surveillance [242, 243]. Bioinformatic meta-analyses of exome and genome sequencing data from patients treated with ICB have further identified tumour genetic characteristics associated with primary resistance to these immunotherapies [238]. The results indicated that clonal TMB was the strongest predictor of response to anti-PD-(L)1, surpassing the predictive value of total TMB. Additionally, the study identified the alteration of cell cycle regulators, the loss of 9q34 and *CCND1* amplification—leading to accelerated cell cycle progression—as significant determinants of ICB response [238, 244]. Other alterations occurring in aneuploid cells also determine response to ICB, notably LOH in regions that include immune regulatory genes, such as those involved in antigen presentation and cytokine signalling [245]. Such genomic instability may lead to the downregulation of MHC class I, essential for T-cell mediated tumour cell killing, thus facilitating immune escape [246]. In other cases, such as with the previously mentioned deletion of the 9p21 chromosomal region, both cell cycle regulators and actors of the tumour cell-autonomous innate immune response are altered, impairing effective immune surveillance [172, 247–249]: this specific genomic alteration has been linked to a “cold” TME, characterized by reduced immune cell infiltration and impaired response to anti-PD-(L)1 agents [247].

Importantly, the frequency and consequences of these mechanisms of resistance can be histotype-specific. For instance, in NSCLC, genomic instability is often driven by tobacco-induced mutagenesis and frequent chromosomal aberrations resulting, amongst others, in mutations in *B2M*, *STK11*, and HLA loss. These alterations impair antigen presentation, interfere with interferon-γ signalling [250, 251], are frequently observed in non-responders to PD-(L)1 blockade and correlate with T-cell exclusion or dysfunction in the TME [189]. In renal cell carcinoma, resistance to anti-PD-1 has instead been associated with a high degree of CIN and the presence of immunosuppressive myeloid populations, including M2-polarized macrophages and neutrophils [252, 253]. Similarly, in some MMRd tumours that are initially sensitive to PD-1 blockade, resistance can be acquired through subclonal mutations in antigen-presentation genes or via immunoediting of immune-resistant clones [254]. In HGSOC, despite encouraging preclinical evidence and in contrast with what has been observed in combination with PARPi, neither *BRCA1/2* mutations nor the HRD status have eventually been significantly associated with improved clinical benefit on anti-PD(L)1 therapy as a monotherapy [255, 256], as exemplified by the absence of difference in progression-free survival (PFS) upon atezolizumab in the phase III IMagyn050 trial (PFS of 21.8 months in the *BRCA1/2*-mutant population vs 18.7 months in the *BRCA1/2*-wildtype population; hazard ratios of 0.87 [95% CI, 0.59–1.29] vs 0.85 [95% CI, 0.70–1.02], respectively) [257]. In that latter trial, most tumours exhibited low TMB and MSI-high status was extremely rare, thereby limiting neoantigen-driven T-cell responses. Furthermore, while a trend towards benefit was noted in *BRCA1*-mutant cases, no significant improvement was observed in *BRCA2*-mutant or HRD-positive tumours, suggesting that, in HGSOC, immunological consequences of genomic instability must be interpreted within a broader, integrated immunological and genomic context to accurately predict benefit from ICB. These examples of histotype-specific patterns illustrate the complexity of immune evasion mechanisms in genomically unstable tumours and underscore the need to incorporate additional parameters beyond TMB, CIN, or genomic instability to better predict response to ICB.

### Challenges in the use of genome instability-related biomarkers in the clinic

Although TMB has been associated with response to ICB across multiple cancer types, histotype-specific characteristics need to be taken into account for reliable patient stratification. For example, a pan-cancer study has shown that TMB-H and HRD were associated with response to ICB in colorectal carcinoma, but not in melanoma or NSCLC [258]. Also, not all DDR defects are associated with high TMB, and the magnitude of overlap between these two features depends on the nature of the DDR alteration [259]; in this context, the use of genomic signatures [260] may be more informative. Lastly, finding the optimal cut-off to discriminate patients with high vs. low TMB also remains a challenge and may be histotype-dependent, which raises major challenges notably for “basket” trials and implementation of clear guidelines in routine clinical practice [261].

While some biomarkers related to HRD are now fully implemented in the clinic, such as *BRCA1/2* mutations in HGSOC [262], others—such as RAD51 foci formation, a biomarker of HRR functionality that enables to assess HRD whatever the underlying aetiology—are not yet fully developed to a clinical grade [263, 264]. To be used as predictors of synthetic lethal interactions, biomarker alterations (such as *BRCA1/2* mutations for PARPi sensitivity or *ATM* loss for ATRi sensitivity) need to be biallelic, clonal, and driver in the disease biology [265]. In this context, using germline or somatic *BRCA1/2* mutations as HRD biomarkers may be challenging, especially in the case of liquid biopsies where clonality assessment is difficult, or when two different monoallelic alterations are combined [266]. Histotype specificities may further complexify biomarker development: for example, HRD positivity is now a biomarker used in standard-of-care for first-line HGSOC, where it has been correlated with overall survival and PFS benefit for patients treated with PARPi as a maintenance therapy [267–270]. However, HRD assays are not clinically used or validated for other tumour types, and these tests are mostly focused on the detection of mutations in HRR genes—thereby missing some other causes of HRD such as *BRCA1* promoter hypermethylation, which is present in 11% of ovarian cancer and 13–25% of breast cancers [271]. Also, HRD status sometimes corresponds to a genomic scar that reflects past exposures to DDR-based therapies, or which has been maintained despite acquisition of resistance to PARPi in HRD tumours; it therefore does not perfectly reflects the current HRR capacity of a patient’s tumour, which limits the positive predictive value of this biomarker for patient selection in clinical trials [272].

## Combination of DNA damage response inhibitors and immune checkpoint blockers: where are we in the clinic?

### Combinations of PARP inhibitors and immune checkpoint blockers

PARP inhibitors (PARPi) mostly act through PARP1 trapping, which stalls replication forks and causes synthetic lethality in HR-deficient tumours, such as BRCA1/2-defective cancers [273, 274]. In such genetic contexts, PARPi also cause the accumulation of micronuclei and induce the cell-autonomous expression of PD-L1, thereby providing a rationale for combining them to anti-PD-(L)1 ICB [15]. The combination of PARPi and ICB in the clinic (Table 1) has first been evaluated in patients with ovarian cancer, in the advanced maintenance setting where PARPi were already approved as a monotherapy. The MEDIOLA ovarian study evaluated the association of the anti-PD-L1 durvalumab and PARPi olaparib in heavily pretreated patients with platinum-sensitive (*n* = 5) or resistant (*n* = 30) ovarian cancer, including six germline *BRCA1/2* (g*BRCA*)-mutant patients. The combination was well tolerated, the most frequent grade 3–4 adverse event being anaemia in 26% of patients. It led to an overall response rate (ORR) and disease control rate (DCR) of 15 and 53%, respectively [275, 276]. In the TOPACIO phase 1/2 study, the association of anti-PD-1 pembrolizumab and PARPi niraparib achieved 65% overall response rate (ORR) in patients with somatic *BRCA1/2*-mutant ovarian cancer, versus 25% in the unselected population (25%), the most common treatment related grade 3–4 adverse event being anaemia (19%) and thrombocytopenia (9%) [277]. Subsequent studies evaluated the triplet combination of a PD-(L)1 inhibitor, PARPi, and VEGFR blockade in the advanced setting, with variable results. The phase II OPAL trial assessed the efficacy of anti-PD-1 dostarlimab, niraparib, and anti-VEGFR bevacizumab in PARPi-naïve patients. The most common grade ≥ 3 adverse events were hypertension (22%) and thrombocytopenia (7.3%). A promising DCR of 76.9% was reached which, importantly, was irrespective of *BRCA1/2*-gene or HRD status [278]. If the GINECO BOLD study also showed that the combination of durvalumab, olaparib, and bevacizumab was safe, it conversely reported a higher DCR in the platinum-resistant compared to the platinum-sensitive population (70% vs 30% at 3 months, respectively) [279]. In a subsequent small phase 1 study that evaluated the association of durvalumab, olaparib, and VEGFR inhibitor cediranib in 9 patients with gynaecological malignancies or triple-negative breast cancer, an ORR of 44% was achieved, and PD-L1 expression correlated with clinical benefit [280]. This was subsequently confirmed in the Phase II OPEB-01 study, which evaluated pembrolizumab, olaparib, and bevacizumab as first-line maintenance in *BRCA1/2*-wildtype patients; the 6-month DCR was 89%, and both HRD and PD-L1 positivity were significantly associated with response [281]. Most recently, the randomized phase III DUO-O study reported a significant PFS benefit in patients receiving durvalumab, olaparib, and bevacizumab, as compared to bevacizumab monotherapy in first-line maintenance (hazard ratio, 0.49 [95% CI, 0.34–0.69]; *P* < 0.0001), regardless of HRD status [282]. Phase 3 registration trials (NCT03602859, NCT03737643, NCT04015739) evaluating such triplet combination in advanced ovarian cancer are being conducted, which will help validating which DDR- and/or immuno-oncology-based biomarkers might be most relevant to select patients.

**Table 1:** Ongoing clinical trials evaluating a combination of DNA damage response inhibitors and immune checkpoint blockers

Study ID	Phase	Study population	Biomarker driven	Study ID
PARPi + ICB				
CVL218 + toripalimab	1/2	All solid tumours	No	NCT06078670
Durvalumab + olaparib or cediranib	1/2	Breast, gynaecological, gastro-intestinal (GI), genito-urinary (GU)	No	NCT02484404
Talazoparib + natural killer cell therapy	1/2	Haematological	No	NCT05319249
Olaparib + durvalumab	2	All solid tumours	Yes (IDHm)	NCT03991832
	2	Lung	Yes (EGFRm)	NCT04538378
Olaparib + pembrolizumab	2	Breast	Yes (gBRCAm or HRD+)	NCT03025035
	2	GI	Yes (gBRCA)	NCT04548752
	2	GI	Yes (TMB-H)	NCT05093231
	2	GI	No	NCT04753879
	2	HNSCC	No	NCT05366166
	2	Gynaecological	No	NCT04483544
	2	Sarcoma	Yes (TLS+)	NCT06116578
	2	Dermatological	Yes (HRR pathway gene mutation)	NCT04633902
Olaparib + magrolimab	2	Breast, GU	Yes (BRCAm)	NCT05807126
Niraparib + dostarlimab	2	All solid tumours	No	NCT05700721
Niraparib + HX008	2	Breast	Yes (gBRCAm)	NCT04508803
Fluzoparib + camrelizumab	2	Lung	No	NCT04782089
	2	HNSCC	No	NCT04978012
PARPi (undisclosed) + pembrolizumab	2	Lung	No	NCT05392686
PARPi + ICB + Radiotherapy				
Olaparib + durvalumab + radiotherapy	1	GI	No	NCT05411094
Niraparib + dostarlimab + radiotherapy	2	Breast	No	NCT04837209
	2	HNSCC	No	NCT04681469
Fluzoparib + camrelizumab + radiotherapy	2	Sarcoma	No	NCT06074692
Pembrolizumab + radiotherapy +/- olaparib	2	GU	No	NCT05568550
PARPi + ICB + Chemotherapy				
Olaparib + pembrolizumab + paclitaxel	2	GI	No	NCT04209686
Niraparib + temozolomide + atezolizumab	2	Lung	No	NCT03830918
Dostarlimab + bevacizumab +/- niraparib +/- paclitaxel	2	Gynaecological	Yes	NCT05065021
Fuzoparib + camrelizumab + temozolomide	1/2	Dermatological	Yes (HRR pathway gene mutation)	NCT05983237
PARPi + ICB + Chemotherapy + Radiotherapy				
Sugemalimab+ olaparib + chemotherapy + radiotherapy	1	Lung	Yes (SLFN-11+)	NCT06217757
Olaparib + durvalumab + carboplatin + etoposide +/- radiotherapy	1/2	Lung	No	NCT04728230
Niraparib + dostarlimab + (chemo)radiotherapy	1/2	HNSCC	No	NCT05784012
ATRi + ICB				
M1774 + avelumab	1	All solid tumours	No	NCT05396833
	2	Dermatological	No	NCT05947500
M1774 + cemiplimab	1/2	Lung	No	NCT05882734
Ceralasertib + Olaparib or durvalumab	2	All solid tumours	No	NCT03682289
Ceralasertib + durvalumab	2	Lung	No	NCT05941897
ATRi + ICB + Chemotherapy				
Ceralasertib (priming) then durvalumab + chemotherapy	2	Breast	No	NCT05582538

Beyond ovarian cancer, combination of PARPi and PD-(L)1 inhibitors has also been investigated in other tumour types, including bladder cancer as neoadjuvant treatment [283], platinum-sensitive CRPC [284], small cell lung cancer [285, 286], and breast cancer [276, 277, 287] (Table 1). In g*BRCA*-mutant breast cancer, while the durvalumab + olaparib combination seemed promising in the MEDIOLA trial, with a 80% DCR at 12 weeks [276], results of the randomized phase II trial comparing anti-PD-L1 atezolizumab plus olaparib versus olaparib monotherapy showed no significant difference in PFS or overall survival [288]. By contrast and as observed in ovarian cancer, a subset of patients derived clinical benefit of this combination irrespective of *BRCA1/2*-gene or PD-L1 status [289], again stressing the need to identify specific biomarkers of response for such combinations. In the JAVELIN tumour-agnostic study, which evaluated the combination of anti-PD-L1 avelumab and PARPi talazoparib in patients with *BRCA1/2* or *ATM* pathogenic alterations, ORR was 26.4%, thereby not meeting the prespecified target ORR of 40% [290, 291]. In this study, higher TMB seemed to be associated with responses, calling for further investigations.

Several studies evaluated the combination of a PARPi with an anti-CTLA-4 antibody, with overall promising results [292–294] (Table 1). Notably, a randomized phase 1b/2 trial performed in 91 patients with advanced pancreatic cancer who had not progressed after 16 weeks of platinum-based chemotherapy, evidenced a 6-month PFS of 59.6% ([95% CI, 44.3–74.9]; *p* = 0·045) in the anti-CTLA-4 ipilimumab + niraparib group, as compared to 20.6% ([95% CI, 8.3–32.9]; *p* = 0.0002 vs. the null hypothesis of 44%) in the anti-PD-1 nivolumab + niraparib group, supporting further evaluation of the former combination.

### Combination of ATR inhibitors and immune checkpoint blockers

ATR inhibitors (ATRi) mediate their anti-tumour activity by forcing tumour cells to enter into mitosis, subsequent to the impairment of the ATR-dependent surveillance of the G2/M cycle checkpoint. Fewer studies have evaluated the association of anti-PD-(L)1 and ATRi (Table 1), but interestingly, ATRi have mainly been used as immunomodulators in such studies—rather than as synthetic lethal partners, to potentially reverse resistance to ICB. In patients with anti-PD-(L)1-resistant or -refractory melanoma, the combination of durvalumab and ATRi ceralasertib achieved a 31% ORR and 63% DCR. Nearly half (46.7%) of patients required a ceralasertib dose reduction due to grade 3/4 anemia (33.3%), thrombocytopenia (16.7%) and neutropenia (16.7%). Transcriptomic profiling suggested that tumours with an immunologically “hot” TME or DDR alterations were more likely to respond [295]. Similar results were observed in the HUDSON trial, which enrolled patients with advanced NSCLC: a 12-week DCR of 61% was observed in the durvalumab + ceralasertib arm, compared to 37% in the durvalumab + olaparib arm [296, 297]. Translational correlates suggested that ceralasertib induced a peripheral increase of the interferon signature, a potential reversion of T-cell exhaustion, and that the addition of durvalumab increased the number of newly expanded T-cell clones. These results were consistent with pre-clinical data showing that intermittent treatment with ceralasertib induced a decrease of intra-tumoural CD8 + and CD4 + T-cells, and the activation of a type I interferon response causing PD-L1 upregulation and tumour sensitization to the combination therapy [54]. This immunomodulatory activity of ceralasertib was further supported by results of the exploratory analysis of the PATRIOT trial, which evaluated this ATRi as a monotherapy in solid tumours: ceralasertib was found to increase the peripheral CD8 +/Treg ratio, granulocytic MDSCs, and CCL2 levels [298].

### Determinants of response and resistance to combinations of DNA damage response inhibitors and immune checkpoint blockers

In line with preclinical data described above, sensitivity to combinations of DDR agents and ICB significantly differs between tumour types, thereby highlighting the role of intrinsic tumour biology, microenvironment, and the importance of appropriate predictive biomarkers. In the MEDIOLA trial (Table 1), patients with ovarian carcinoma who received the combination of durvalumab and olaparib presented a significant difference in ORR depending on the presence (ORR 92.2% [95% CI, 81.1–97.8]) or absence (ORR 34.4% [95% CI, 18.6–53.2]) of a g*BRCA* mutation. Median duration of response was also better for patients with g*BRCA* mutation (14.8 months [9.0–not reached] vs 6.9 months [5.4–11.1]) [299]. In another trial evaluating the triple combination of durvalumab, olaparib, and fulvestrant in hormonal receptor-positive, HER2-negative breast cancer patients, the presence of a *BRCA1/2* mutation was also linked to a benefit in PFS (12.6 months [95% CI, 8.2–16.7]) vs 9.0 months [95% CI, 6.7–11.2]) compared to *BRCA1/2*-wildtype patients [300]. By contrast, in platinum-resistant HGSOC, the combination of bevacizumab, olaparib, and durvalumab was associated with a worse 3-month PFS for patients with *BRCA1/2*-mutant tumours compared to those with *BRCA1/2*-wildtype tumours (50.0% [90% CI, 10.3–80.9] vs 72.2% [90% CI, 57.8–82.4]); in line with this finding, patients who had previously received PARPi had a lower PFS, thereby suggesting a role for acquired resistance to PARPi in the worse outcome of this patient population, despite the triple combination therapy. By contrast, TME-related biomarkers, notably a higher tumour inflammation signature, were associated with better outcome [301]. When comparing all study results in HGSOC, there is currently no consistent pattern regarding the best predictive biomarkers for DDRi plus ICB combinations (Table 1). While *BRCA1/2*-mutated or HRD status were identified as predictive biomarker of response and better outcome in the MEDIOLA (olaparib, durvalumab) [299], DUO-O (durvalumab, olaparib and bevacizumab) [282], and OPEB-01 (pembrolizumab, olaparib, bevacizumab) [281] trials, it was not the case in the TOPACIO (pembrolizumab, niraparib) [289], OPAL (dostarlimab, niraparib, bevacizumab) [278], and GINECO BOLD (durvalumab, olaparib, bevacizumab) studies [301]. Similarly, PD-L1 positivity was associated with better outcome in the OPEB-01 study [281] but not in MEDIOLA [299], TOPACIO [289], and OPAL [278] trials.

In NSCLC, the combination of olaparib and durvalumab showed a poorer median PFS in patients with mutations in HRR genes, compared to those without (3.9 months [95% CI, 1.8–7.5] vs 7.4 months [95% CI, 5.5–9.3]), while PD-L1 expression greater than 50% was associated with a better PFS (10.1 months [95% CI, 3.7–not reached] vs 5.7 months [95% CI, 2.8–8.8] in patients with PD-L1-null tumours). This suggests that HRD status may not be a useful predictor of the benefit of PARPi plus anti-PD-L1 therapy, at least in NSCLC, or that induction platinum exposure may have confounded this effect. Interestingly, patient with non-squamous histology also tended to derive a greater median PFS benefit, again potentially suggesting histotype-specific dependencies [302].

In the JAVELIN PARP Medley trial (Table 1), which evaluated the combination of avelumab and talazoparib in several cancer types including breast, ovarian, prostate, urothelial carcinoma, or NSCLC, alterations in DDR genes were not associated with response for patients with urothelial carcinoma or NSCLC, reinforcing the observation that DDR genes alterations play a key role in the biology of some cancers but are more neutral in others [291]. A study evaluating the combination of rucaparib and atezolizumab in TNBC and gynaecological cancers with DDR gene alterations also showed that PD-L1 expression and a high CD8 + T-cell infiltration at baseline were associated with better outcome on treatment, suggesting a relationship between a pre-existing immune response and benefit on therapy. Paired biopsies performed on patients during treatment also showed an increase of PD-L1 expression, CD8 + T-cell infiltration, and cGAS-STING pathway activation only in responders [303]. Similar findings were recently reported from the ARIANES trial which also evaluated the rucaparib plus atezolizumab combination but in other tumour types [304, 305]: single-cell analyses performed sequentially on tumour biopsies of one responding g*BRCA*-mutant CRPC patient suggested that cGAS-STING activation occurred both in tumour cells and in some T-cell subpopulations, as suggested preclinically [15]. Translational analyses from the MEDIOLA trial also found an increase of CD8 + T-cell and NK cell infiltration, and a decrease of myeloid cell infiltration upon olaparib treatment, which was further increased when combining durvalumab; preclinically, these changes were only seen in *Brca1*-mutant BR5 mouse models and not in *Brca*-wildtype models and were associated with sensitivity to treatment [306]. In the TOPACIO study, which evaluated the combination of niraparib and pembrolizumab in patients with ovarian carcinoma, single-cell analyses concluded that HRD status (as assessed by the “Sig3” specific mutational signature that also takes into account large deletions), CD8-exhausted effector T-cells in the tumour microenvironment and activation of the interferon pathway could be used as surrogate biomarkers of response [218].

Regarding the ATRi plus ICB combination, patients with NSCLC treated with ceralasertib and durvalumab in the HUDSON study (Table 1) had a better ORR and PFS when *ATM* was mutated (ORR of 26.1%, median PFS of 8.4 months) compared to those with *ATM*-wildtype tumours (ORR of 13% and 6.1% in patients with primary or acquired resistance to ICB, respectively; median PFS of 4.6 months) or acquired resistance to ICB without any biomarker (ORR of 6.1%, median PFS of 4.6 months). No difference was observed according to PD-L1 status [307]. Interestingly, the same combination evaluated in patients with advanced melanoma showed no difference in terms of response according to ATM or PD-L1 status; patients with HRD tumours seemed to benefit more from this combination, although no significant difference in PFS could be observed (hazard ratio, 0.17 [95% CI, 0.02–1.43]). Higher expression of Tregs (*p* = 0.02), interferon genes (*p* = 0.03), and MHC class I (*p* = 0.003) signatures was observed in responders [295]. Finally, in the PATRIOT trial, response was associated with an inflamed TME at baseline [298].

Altogether, this data strongly supports that, beyond histotype, both tumour molecular features and microenvironment characteristics influence response to treatment and should be considered when selecting patients for treatment with a combination of DDR agents and ICB.

### New kids on the block for future combinations

Future combinations with ICB will soon emerge as new DDR-targeting drugs are being developed [308].

Similar to ATRi, WEE1 inhibitors (WEE1i) force progression of tumour cells through the G2/M cell cycle checkpoint, thereby favouring micronuclei formation and genomic instability when DNA lesions are not adequately repaired. A phase 1 trial of the first-in-class WEE1i adavosertib reported a 14% ORR in biomarker-unselected patients, although CCNE1 overexpression seemed to be associated with response [309]. Since WEE1 inhibition promotes the activation and recruitment of immune cells by inducing an interferon-γ response [310], combination with immune-based therapies are already being evaluated in a preclinical setting [311].

CHK1, as the major downstream effector of ATR, has also been tested as a druggable target over the past years. If results of monotherapy CHK1 inhibitors (CHK1i), such as prexasertib or SRA737, have been rather disappointing [312–315], combinations with ICB could be beneficial to patients [316, 317]. Since ATRi, WEE1i and CHK1i act on the same cell cycle checkpoint but through distinct mechanisms, it will be interesting to later investigate similarities and differences in their immunomodulatory effects.

PARP1-selective PARPi, currently under development to lower PARP2-mediated haematologic toxicity, could harbour very interesting immunomodulatory capabilities [318]. Notably, a phase 1 trial evaluating the PARP1-selective PARPi AZD5305 in patients with DDR defects showed very promising results, with a very favourable safety profile and a higher exposure than first-generation PARPi [319, 320]. Based on their mechanism of action and lower haematological toxicity, we can expect that the combination with ICB will bring higher benefit than that of first-generation PARPi [321].

Other compounds currently in the clinic that have not yet been evaluated in combination with immune-based therapies include PKMYT1, USP1, and DNA-PK inhibitors [308]. PKMYT1 (membrane-associated tyrosine- and threonine-specific CDC2-inhibitory kinase) is a negative regulator of CDK1 which has shown activity in tumours with *CCNE1* amplification, *FBXW7* and *PPP2R1A* mutations [322] and is already being evaluated in combination with other compounds such as chemotherapy [323]. USP1, a deubiquitinase involved in the DDR by modulating ubiquitination of a number of HRR regulators such as FANCD2, is highly expressed in cancers and may represent a new important target to reverse resistance to PARPi in *BRCA*-mutant tumours [324]. Since USP1 also plays a role in Th17 and Treg differentiation, combination with ICB may bring additional benefit [325]. Finally, DNA-PK is a member of the PI3K family that plays a role in the repair of DSBs via NHEJ. DNA-PK inhibitors have been evaluated as a monotherapy and in combination with radiotherapy in HNSCC and rectal cancer, with overall disappointing results [326–329]. Still, novel small molecules, such as triple DNA-PK inhibitors that also affect the immunomodulatory isoform PI3Kδ/γ, thereby favouring CD8 + cytotoxic T-cell increase and Tregs depletion in mice models, may represent interesting alternatives for combinations which ICB [330].

## Conclusion and perspectives

There is now compelling preclinical and clinical evidence that the extensive crosstalk between genome instability, DDR, and anti-tumour immunity plays a central role in carcinogenesis, tumour progression and response to treatment. If most attention has initially focused on the cGAS-STING DNA sensing pathway and PD-(L)1 blockers, subsequent complementary work has shown that other important signalling mechanisms, including cytosolic RNA or RNA:DNA hybrids sensing, aneuploidy, or RNA editing, also play a key role in this interplay. Most importantly, the recent description of reciprocal feedback loops (nucleus-to-cytoplasm and cytoplasm-to-nucleus signalling) involving actors of the innate immunity pathways, RSR and DDR, illustrates the dynamic characteristic of this crosstalk and highlights its complexity.

Beyond these significant advances in preclinical mechanistic understanding, clinical successes based on strategies that exploit the genome instability–anti-tumour immune response interplay have started to be achieved. If initial results of the multiple studies evaluating the PARPi plus anti-PD-(L)1 combinations have been mitigated (Table 1) [2, 331], they have shown case, in patients, that DDR inhibitors can activate cell-autonomous innate immune pathways in cancer cells [305, 332], and intriguing responses observed in HR-proficient, PD-L1-negative patients call for further translational research to best identify predictive biomarkers of such combination [285]. Furthermore, recent successes obtained notably when combining ATR inhibitors with anti-PD-(L)1 open promising perspectives, especially for combinations with agents that are more potent than PARPi in inducing replication stress [307]. The multiple novel, selective and potent DDR inhibitors that are currently entering early phase trials have almost all been reported to display – at least preclinically – immunogenic properties through various mechanisms, but whether this will translate into tangible clinical benefit is still unknown. Appropriate patient selection, optimal choice of the drug to be combined with – notably beyond anti-PD-(L)1, scheduling and disease setting, still need to be defined. In this regard, forthcoming clinical trials that will evaluate intermittent dosing of DDR inhibitors may bring interesting results. Similarly, combinatorial patient selection biomarkers that would include both DDR, replication stress and immune-based biomarkers, may allow to best identify patients who may benefit from such combinations.

Finally, newer classes of agents may represent relevant candidates for therapeutic combination strategies that exploit the genome instability–anti-tumour immune response interplay. In particular, antibody–drug conjugates have shown impressive recent results in combination with anti-PD-(L)1 therapy in multiple tumour types (e.g. enfortumab vedotin + pembrolizumab in bladder cancer [333], or various combinations in NSCLC [334]) and currently represent one of the most promising therapeutic avenues in cancer therapy. Alongside appropriate translational research to fully understand the mechanism of action of these combinations in patients [305, 335] and identify the best selection biomarkers, this will foster the development of novel, effective therapeutic strategies for patients’ benefit.

## Data Availability

No datasets were generated or analysed during the current study.
